# Elucidating the influences of social determinants of health on perceived overall health among African American/Black and Hispanic ovarian cancer survivors using the NIH *All of Us* Research Program

**DOI:** 10.1016/j.ygyno.2024.06.027

**Published:** 2024-07-09

**Authors:** Diane E. Mahoney, Rishav Mukherjee, Jeffrey Thompson

**Affiliations:** a School of Nursing, University of Kansas Medical Center, Kansas City, KS 66160, USA; b Department of Biostatistics and Data Science, University of Kansas Medical Center, Kansas City, KS 66160, USA

## Abstract

**Objectives.:**

To evaluate the influences of social determinants of health (SDOH) on perceived health and well-being among African American (AA)/Black and Hispanic ovarian cancer survivors.

**Methods.:**

A cross-sectional study was conducted using overall health and SDOH survey data collected by the National Institutes of Health *All of Us* Research Program from May 2017 to September 2023.

**Results.:**

While 1250 enrolled participants with ovarian cancer met the inclusion criteria, 414 (33%) completed SDOH surveys: 29 (7%) AA/Black, 33 (8%) Hispanic, and 352 (85%) White. In the ordinal logistic regression models, for every unit increase in the SDOH neighborhood characteristics score, the odds of having a poor perception of general health decreased by 0.96 times. For every unit increase in the SDOH day-to-day discrimination score, the odds of having a poor perception of general health, general mental health, social satisfaction decreased by 0.95, 0.94 and 0.93 times respectively. For every unit increase in the SDOH food and housing security score, the odds of having a poor perception of general health decreased by 0.57 times. Compared to White ovarian cancer survivors, AA/Black and Hispanic ovarian cancer survivors were significantly more likely to have a poor perception of general health, general mental health, and social satisfaction even when adjusting for these SDOH.

**Conclusions.:**

Unfavorable SDOH conditions negatively influence the overall perception of health. These findings signal an urgency for healthcare professionals and scientists to partner together with local communities in designing feasible and imaginative interventions to overcome cancer care disparities in an equitable manner.

## 1. Introduction

Nearly 20,000 women are diagnosed with ovarian cancer in the United States (US) annually, and over 13,000 women die of the disease [1]. Although there are no proven effective methods to screen for early disease and the incidence of ovarian cancer is higher in White women, African American/Black and Hispanic women continue to experience disparities in survival and recurrence rates [2–8]. The overall 5-year survival rate for African American/Black women is 41% and 23% when diagnosed at late stage compared to 49% and 34% among White women [1]. Furthermore, African American/Black women are more likely to be diagnosed with advanced disease and more aggressive ovarian tumors [8,9]. Even though Hispanic women are diagnosed at an earlier stage on average, they tend to be younger compared to White women, and inequitable access to health care services remains an ongoing challenge. Sadly, research has shown when healthcare access is equal between White and African American/Black women when adjusting for neighborhood-level socioeconomic status covariates, ovarian cancer treatment outcomes remain worse for African American/Black women [2,10,11]. While the root causes of ovarian cancer disparities are multidimensional, social determinants of health (SDOH) are considered key contributors.

SDOH are powerful driving forces of ovarian cancer disparities because they encompass the places where women are born, live, grow, and age as they navigate within the healthcare system. Structural racism is shown to affect delivery of cancer care [12]. Oncologist implicit racial bias is negatively correlated with oncologist communication toward patients, reactions of patients to racially discordant oncology interactions, and patient confidence and adherence to recommended treatments [13]. African American/Black and Hispanic women with ovarian cancer are shown to be less likely to receive timely treatment with surgery and chemotherapy [10,14]. Healthcare access barriers to African American/Black and Hispanic women include receipt of a gynecologic oncology consult, type of health insurance status, proximity to high volume cancer centers, receipt of adequate patient education, and the absence of National Comprehensive Cancer Network guideline adherent cancer care [14–17]. Thus, ovarian cancer survivors belonging to underrepresented racial and ethnic groups are vulnerable to mistrust of health professionals and medical treatment, which may heighten the underrepresentation of African American/Black and Hispanic women with ovarian cancer in clinical trials.

African American/Black and Hispanic women report having lower levels of emotional support (i.e. having someone to talk with, assistance with chores) during gynecologic cancer evaluation and treatment compared to White women [18]. Nonetheless, there are knowledge gaps in understanding self-perception of overall health and well-being among ovarian cancer survivors who are African American/Black and Hispanic. Self-perception of health can mirror the capability to function in a definite social structure and organizational system (such as the healthcare system) and is considered a prognostic indicator of chronic disease prevalence [19]. In African American/Black women, for example, health-related quality of life (HRQOL) has been significantly associated with social support, anxiety, poverty, and perceived discrimination [20]. Therefore, elucidating African American/Black and Hispanic women’s perceptions of health will be essential for addressing unmet needs throughout ovarian cancer survivorship. The objective of this study is to evaluate the influences of SDOH on perceived health and well-being among African American/Black and Hispanic ovarian cancer survivors. Our research question is, “*What is the relationship between SDOH and self-perceptions of overall health among ovarian cancer survivors who are African American/Black, and Hispanic compared to those who are White?*” We hypothesized that certain SDOH variables will negatively influence perceptions of general health, mental health, and social satisfaction among African American/Black and Hispanic ovarian cancer survivors.

## 2. Material and methods

### 2.1. Participants and data source

Data generated from participants enrolled in the National Institutes of Health *All of Us* Research Program was used in this study. *All of Us* is a precision health initiative that integrates a longitudinal cohort study of historic efforts to actively collect data directly from racially and ethnically diverse individuals and from their electronic health records [21]. A major goal of the *All of Us* Research Program is to obtain study data from one million or more individuals who are representative of those living in the US. >500,000 participants are currently enrolled, 80% of whom are underrepresented populations [22].

### 2.2. Design

A cross-sectional study was conducted using overall health and SDOH survey data collected by the *All of Us* Research Program from May 2017 to September 2023. Study inclusion criteria were: (1) malignant tumor of ovary as a medical condition as individuals are considered cancer survivors from the time of diagnosis through the balance of life until the end of life [23]; (2) age 18 years or older; (3) assigned female at birth; and (4) African American/Black, Hispanic, and White race and/or ethnicity. Individuals who did not complete SDOH study surveys were excluded from the analysis.

### 2.3. Statistical analysis

Ordinal logistic regression models were fit to the *Overall Perception of Health survey* data to assess their associations with SDOH survey elements. To determine how these effects changed across the race categories (White and African American/Black) and ethnicities (Hispanic and Latinos vs non-Hispanic and Latinos), we included them as dichotomous covariates in the model. “White” was the race category of reference and “non-Hispanic and Latino” was the reference category for ethnicity. Based on individual responses, a numerical score was attached to each category with the highest score being attributed to the most positive response and the lowest to the most negative response from the perspective of the question or domain. As an example in case of scoring patient response based on the SDOH domain pertaining to day-to-day discrimination the following were the scores associated with each response:

- Never – 5
- Less than once a year – 4
- A few times a year – 3
- A few times a month – 2
- At least once a week – 1
- Almost every day – 0

Additional details of scoring are provided in the Supplementary materials. The scores were then added across the domains of the question that was considered. Thus, a unit increase in the SDOH score for a domain would indicate incrementally better social conditions for the given responder. Models were fit to assess the associations between domains of overall perception of health and SDOH as described in Table 1.

All the models defined in Table 1 were adjusted for dichotomous covariates of key demographic factors, namely race and ethnicity, to investigate the disparities in perception of overall health. While specific SDOH (neighborhood characteristics, day-to-day discrimination, food and housing security, supportive relationships, and spiritual life) were the variables of interest, we adjusted for race and ethnicity in our models to evaluate if overall health perception differences by race and ethnicity would persist beyond the SDOH assessed in this study. Only data from study participants who completed all the SDOH surveys were assessed. Odds ratios (OR) along with their respective 95% confidence intervals were calculated of the outcome for study participants in the datasets to have a poor perception of overall health as compared to all the other levels (Fair, Good, Very Good and Excellent). ORs were calculated from the contrasts of the previously selected reference groups. All statistical tests were two sided, and a *p*-value <0.05 was considered to be statistically significant. No multiplicity adjustments were made.

## 3. Results

Of the total of 1250 *All of Us* enrolled participants with ovarian cancer, 414 (33%) completed the SDOH surveys. Of those, 29 (7%) African American/Black, 33 (8%) Hispanic, and 352 (85%)White participants were included in the analysis. Participant demographic characteristics are presented in Table 2.

### 3.1. General health perception

We evaluated five SDOH domains (neighborhood characteristics, discrimination in day-to-day life, supportive relationships, food and housing security, and spiritual life) from the SDOH survey. Neighborhood characteristics survey items included questions such as: (1) *How much do you agree or disagree that in your neighborhood people watch out for each other?;* (2) *How much do you agree or disagree that your neighborhood is noisy?;* (3) *How much do you agree or disagree that your neighborhood has a lot of drug use?;* (4) *How much do you agree or disagree that your neighborhood is clean?;* and (5) *How much do you agree or disagree that there is a lot of crime in your neighborhood?* Participant response options (Strongly Disagree, Disagree, Neutral, Agree, Strongly Agree) were converted to numerical scores based on their responses. For example, in case of the question, *How much do you agree or disagree that your neighborhood has a lot of drug use?*, the scores were assigned as Strongly Disagree – 4, Disagree – 3, Neutral – 2, Agree – 2, Strongly Agree – 0. On the other hand for questions such as *How much do you agree or disagree that your neighborhood is clean?*, the scores were assigned as Strongly Agree – 4, Agree – 3, Neutral – 2, Disagree – 2, Strongly Disagree – 0. Higher scores represent better neighborhood conditions. Questions that evaluated general health perception included statements such as: (1) *In general, would you say you heath is;* (2) *In general, would you say your quality of life is;* and (3) *In general, how would you rate your physical health?* General health perception survey responses levels were rated as Poor, Fair, Good, Very Good, and Excellent. For every unit increase in the SDOH neighborhood characteristics score, the odds of having a poor perception of general health (versus all other levels) decreased by 0.96 times. The odds of having a poor perception of general health were 2.48 times higher for African American/Black ovarian cancer survivors compared to those who are White when adjusting for neighborhood characteristics and ethnicity. The odds of having a poor perception of general health were 2.86 times higher among Hispanic ovarian cancer survivors compared to those who are non-Hispanic when adjusting for neighborhood characteristics and race.

Day-to-day discrimination survey items included statements such as (1) *You are treated with less courtesy than other people are;* (2) *You are treated with less respect than other people are;* (3) *You receive poorer service than other people at restaurants or stores;* and (4) *People act as if they are afraid of you. Participant response options (Never, Less than once a year, A few times a year, A few times a month, At least once a week, and Almost every day)* were converted to numerical scores as described in the Methods section. Higher scores represent less exposure to day-to-day discrimination. For every unit increase in the SDOH day-to-day discrimination score, the odds of having a poor perception of general health (versus all other levels) decreased by 0.95 times. The odds of having a poor perception of general health were 2.2 times higher for African American/Black ovarian cancer survivors compared to those who are White when adjusting for day-to-day discrimination and ethnicity. The odds of having a poor perception of general health were 3.45 times higher among Hispanic ovarian cancer survivors compared to those who are non-Hispanic when adjusting for day-to-day discrimination and race.

Food and housing security survey items included statements such as: (1)*Within the past 12 months, were you worried whether the food you had bought just didn’t last and you didn’t have money to get more? and* (2) *In the last 12 months, how many times have you or your family moved from one home to another? Participant response options (Often true, Sometimes true, Never true,)* were converted to numerical scores and assigned as Never True – 2, Sometimes True – 1, Often True – 0. Higher scores represent better food and housing security conditions. For every unit increase in the SDOH food and housing security score, the odds of having a poor perception of general health (versus all other levels) decreased by 0.57 times. The odds of having a poor perception of general health were 2.25 times higher for African American/Black ovarian cancer survivors compared to those who are White when adjusting for food and housing security and ethnicity. The odds of having a poor perception of general health were 4.40 times higher among Hispanic ovarian cancer survivors compared to those who are non-Hispanic when adjusting for food and housing security and race.

Spiritual life survey items included questions such as (1) *How often do you feel God’s (or a higher power’s) love for you, directly or through others?;* (2) *How often do you feel that you are spiritually touched by the beauty of creation?;* (3) *How often do you feel God’s (or a higher power’s) presence?;* and (4) *How often do you go to religious meetings or services?* Participant response options were converted to numerical scores and assigned as Many times a day – 9, Every day – 8, Most Days – 7, More than once a week – 6, Some days – 5, Once a week – 4, 1 – 3 times a month – 3,Once in a while – 2, Less than once per month – 1, Never/Almost never / I do not believe in God or a higher power - 0. Higher scores represent a greater perception of spirituality. For every unit increase in the SDOH spiritual life score, the odds of having a poor perception of general health (versus all other levels) decreased by 0.99 times. The odds of having a poor perception of general health were 3.58 times higher for African American/Black ovarian cancer survivors compared to those who are White when adjusting for spiritual life and ethnicity. The odds of having a poor perception of general health were 2.98 times higher among Hispanic ovarian cancer survivors compared to those who are non-Hispanic when adjusting for spiritual life and race. OR values and confidence intervals obtained by models of association between general health perception and SDOH (neighborhood characteristics, day-to-day discrimination, food and housing security, and spiritual life) are provided in Table 3.

### 3.2. General mental health perception

Supportive relationships survey items included questions such as (1) *How often do you have someone to love and make you feel wanted?;* (2) *How often do you have someone to help you if you were confined to bed?;* and (3) *How often do you have someone to turn to for suggestions about how to deal with a personal problem? Participant response options (None of the time, A little of the time, Some of the time, Most of the time, All of the time)* were converted to numerical scores and assigned as All of the time – 4, Most of the time – 3, Some of the time – 2, A little of the time – 1, None of the time – 0. Higher scores represent greater levels of social support. Questions that evaluated general mental health perception included statements such as: (1) *In general, how would you rate your mental health, including your mood and your ability to think?* and (2) *In the past 7 days, how often have you been bothered by emotional problems such as feeling anxious, depressed or irritable?* General mental health perception survey responses were rated as Poor, Fair, Good, Very Good, and Excellent or as Always, Often, Sometimes, Rarely, and Never. For every unit increase in the SDOH supportive relationship score, the odds of having a poor perception of general mental health decreased by 0.96 times. The odds of having a poor perception of general mental health were 1.76 times higher for African American/Black ovarian cancer survivors compared to those who are White when adjusting for supportive relationship and ethnicity. The odds of having a poor perception of general mental health were 8.4 times higher among Hispanic ovarian cancer survivors compared to those who are non-Hispanic when adjusting for supportive relationship and race.

For every unit increase in the SDOH day-to-day discrimination score, the odds of having a poor perception of general mental health decreased by 0.94 times. The odds of having a poor perception of general mental health were 1.4 times higher for African American/Black ovarian cancer survivors compared to those who are White when adjusting for day-to-day discrimination and ethnicity. The odds of having a poor perception of general mental health were 5.8 times higher among Hispanic ovarian cancer survivors compared to those who are non-Hispanic when adjusting for day-to-day discrimination and race. For every unit increase in the SDOH spiritual life score, the odds of having a poor perception of general mental health decreased by 0.97 times. The odds of having a poor perception of general mental health were 3.3 times higher for African American/Black ovarian cancer survivors compared to those who are White when adjusting for spiritual life and ethnicity. The odds of having a poor perception of general mental health were 5.26 times higher among Hispanic ovarian cancer survivors compared to those who are non-Hispanic when adjusting for spiritual life and race. OR values and confidence intervals obtained by models of association between general mental health perception and SDOH (supportive relationships, day-to-day discrimination, and spiritual life) are provided in Table 4.

### 3.3. Social satisfaction perception

Questions that evaluated social satisfaction perception included statements such as: (1) *In general, how would you rate your satisfaction with your social activities and relationships?* and (2) *In general, please rate how well you carry out your social roles (this includes activities at home, at work and in your community, and responsibilities as a parent, child, spouse, employee, friend,* etc.). Social satisfaction perception survey responses were rated as Poor, Fair, Good, Very Good, and Excellent. For every unit increase in the SDOH spiritual life score, the odds of having a poor perception of social satisfaction decreased by 0.98 times. The odds of having a poor perception of social satisfaction were 2.67 times higher for African American/Black ovarian cancer survivors compared to those who are White when adjusting for spiritual life and ethnicity. The odds of having a poor perception of social satisfaction were 4.73 times higher among Hispanic ovarian cancer survivors compared to those who are non-Hispanic when adjusting for spiritual life and race. For every unit increase in the SDOH supportive relationship score, the odds of having a poor perception of social satisfaction decreased by 0.94 times. The odds of having a poor perception of social satisfaction were 1.68 times higher for African American/Black ovarian cancer survivors compared to those who are White when adjusting for supportive relationship and ethnicity. The odds of having a poor perception of social satisfaction were 6.9 times higher among Hispanic ovarian cancer survivors compared to those who are non-Hispanic when adjusting for supportive relationship and race.

For every unit increase in the SDOH food and housing security score, the odds of having a poor perception of social satisfaction decreased by 0.53 times. The odds of having a poor perception of social satisfaction were 1.3 times higher for African American/Black ovarian cancer survivors compared to those who are White when adjusting for food and housing security and ethnicity although not statistically significant (*p* = 0.19). The odds of having a poor perception of social satisfaction were 5.63 times higher among Hispanic ovarian cancer survivors compared to those who are non-Hispanic when adjusting for food and housing security and race.

For every unit increase in the SDOH day-to-day discrimination score, the odds of having a poor perception of social satisfaction decreased by 0.93 times. The odds of having a poor perception of social satisfaction were 1.14 times higher for African American/Black ovarian cancer survivors compared to those who are White when adjusting for day-to-day discrimination and ethnicity although not statistically significant(*p* = 0.29). The odds of having a poor perception of social satisfaction were 4.53 times higher among Hispanic ovarian cancer survivors compared to those who are non-Hispanic when adjusting for day-to-day discrimination and race. OR values and confidence intervals obtained by models of association between social satisfaction perception and SDOH (spiritual life, supportive relationships, food and housing security, and day-to-day discrimination) are provided in Table 5. Additional OR values and confidence intervals for associations between race or ethnicity and perceptions of overall health when adjusting for SDOH scores are found in Supplementary Tables 1–6.

## 4. Discussion

In this study, we utilized *All of Us* Research Program data to analyze the relationship between perceptions of overall health, and SDOH among underrepresented racially and ethnically diverse ovarian cancer survivors. Our findings demonstrate that SDOH factors significantly influence self-perceptions of overall health. However, large differences observed by race and ethnicity persisted when adjusting for specific SDOH variables. As ovarian cancer disparities are prevalent within African American/Black and Hispanic populations, there is uncertainty on the magnitude to which negative self-perceptions of general health, general mental health, and social satisfaction described in our findings subsequently impact women’s healthcare utilization and adherence to health provider recommendations. Racial-related symptom disparities have been observed in cancer care within underrepresented racial and ethnic groups that have correlated with poor health outcomes [24–26]. Hispanic women are shown to have a longer time from symptom presentation to diagnosis, and African-American/Black women have a longer time from diagnosis to treatment compared to White women [27]. Racial and ethnicity differences are described in patient navigation from the time a women first reports ovarian cancer symptoms to the time of diagnostic workup and appropriate referrals [28]. In comparison to other racial and ethnic groups, Hispanic cancer survivors have reported higher symptom burden and lower HRQOL [29]. Although African American/Black women are more likely to report clinically meaningful symptoms, they have described these symptoms as not being appropriately addressed when reported to health professionals [30]. Moreover, African American/Black women with ovarian cancer who perceive daily discrimination are more likely to have prolonged symptoms [31]. African American/Black and Hispanic women are more likely to have dose reductions in comparison to Whites, and African American/Black women are more likely to have treatment delay and early treatment discontinuation [32]. Unfortunately, African American/Black patients are shown to have the greatest delays in receiving care across cancer populations [33].

SDOH factors (*discrimination in day-to-day life, food and housing security, spiritual life, and supportive relationships*) assessed in our study had profound influences on ovarian cancer survivors’ general health outlook. SDOH are powerful factors that also impact HRQOL and health outcomes of women with ovarian cancer [34,35]. Our study identified food and housing security and a favorable neighborhood environment as detrimental to women’s sense of general well-being during survivorship. Social characteristics reported by African American/Black cancer survivors have included food and housing insecurity, perceived discrimination, utility shutoffs, lack of transportation, not receiving care due to cost, and neighborhood safety concerns, which lowered HRQOL [20,35]. In the current study, spiritual life was statistically significantly associated with perceptions of general health (*p* < 0.00), general mental health (*p* = 0.00), and social satisfaction (*p* < 0.00) among African American/Black and Hispanic ovarian cancer survivors. One qualitative study found that long-term ovarian cancer survivors attributed their survival and disease coping to medical, social, religious/spiritual, and lifestyle/personal characteristics [36]. Another qualitative study found that economic instability and structural barriers negatively influenced access to care and social well-being among women with recurrent ovarian cancer [37].

Our findings demonstrate that African American/Black and Hispanic ovarian cancer survivors were significantly more likely to have a poor perception of social satisfaction and general mental health compared to women who were White and non-Hispanic. In general, Hispanic individuals with cancer have reported worse social and overall HRQOL, depression, and distress compared to other ethnic minorities [38]. However, electronic health records documentation of distress screening and follow-up psychosocial care is shown to be lowest for Hispanic women with ovarian cancer [39]. Moreover, African American/Black and Hispanic ovarian cancer survivors are less likely to receive antidepressants compared to those who are White [40]. Among African American/Black individuals with cancer, mental health quality of life has increased with higher social support levels [41]. Therefore, patient navigation has a valuable role in cancer care delivery by addressing the SDOH-associated barriers to healthcare access among vulnerable populations [42].

Patient navigation, cancer screening, and the quality of patient-to-provider interactions from time of diagnosis and throughout survivorship remain problematic for African American/Black and Hispanic patients [43–45]. Racial discrimination in healthcare settings is shown to disrupt the patient-clinician relationship, negatively influence health-seeking behaviors, and degrade one’s sense of healthcare system trustworthiness [46]. Thus healthcare institutional investment in genuine efforts to build a deeper trust is strongly suggested within vulnerable communities who traditionally mistrust the healthcare system [42]. Additionally, ongoing development of multi-agency collaborations between community organizations and cancer centers could be beneficial for linking ovarian cancer survivors to services [35]. Workforce diversity expansion has gained national attention to reduce cultural barriers, foster patient satisfaction, promote greater patient trust in provider treatment recommendations, and increase clinical trial participation of underrepresented groups [34,47]. However, African American/Black individuals have specifically identified racism, negative media perceptions, health insurance, health literacy, child care, and transportation issues as barriers to clinical trial enrollment [48].

A major strength of this study is the utilization of *All of Us* Research Program data that incorporate women from racial and ethnic groups who are representative of the US population but largely underrepresented in biomedical research. One study limitation is the modest sample size of African American/Black and Hispanic participants who completed the SDOH questionnaires that could have impacted the generalizability of the findings. Additional limitations to the database including the absence of available data to evaluate factors such as: (1) time of ovarian cancer diagnosis; (2) ovarian tumor histology; (3) tumor stage and grade; (4) prior treatment; (5) recurrent disease status; (6) timepoint to which SDOH surveys were completed in relationship to patient navigation in cancer care. Lastly, we did not assess for all possible SDOH factors, which may have accounted for the persistent differences in overall health perceptions between African American/Black and Hispanic ovarian cancer survivors compared to those who are White when adjusting for the SDOH factors that we evaluated. These observations suggest additional residual effects of additional SDOH that warrant follow-up studies.

## 5. Conclusions

While ovarian cancer disparities are well documented, influences of SDOH on survivors’ perceptions of overall health are largely underexplored. Fundamentally, our study sought to elucidate SDOH domains that impact African American/Black and Hispanic women’s perceptions of general health, general mental health, and social satisfaction using data from the *All of Us* Research Program. While additional SDOH factors should also be explored, our compelling study findings challenge healthcare professionals and scientists to partner together with communities in designing feasible and imaginative interventions to overcome ovarian cancer care disparities in an equitable manner.

## Supplementary Material

Supplementary Tables

Appendix A. Supplementary data

Supplementary data to this article can be found online at https://doi.org/10.1016/j.ygyno.2024.06.027.

## Figures and Tables

**Table 1:** Perception of overall health and social determinants of health association models.

	Overall Health Domain	SDOH Domain Score

Model A1	General Health	Neighborhood Characteristics
Model A2	General Health	Discrimination in Day-to-Day Life
Model A3	General Health	Food and Housing Security
Model A4	General Health	Spiritual Life
Model B1	General Mental Health	Supportive Relationships
Model B2	General Mental Health	Discrimination in Day-to-Day Life
Model B3	General Mental Health	Spiritual Life
Model C1	Social Satisfaction	Supportive Relationships
Model C2	Social Satisfaction	Discrimination in Day-to-Day Life
Model C3	Social Satisfaction	Food and Housing Security
Model C4	Social Satisfaction	Spiritual Life

**Table 2:** Participant demographic characteristics.

	N	%

*Age*		
18 to 44 years	124	9.9
45 to 64 years	515	41.2
>65 years	611	48.8
*Race*		
African American/Black		
Total	144	11.5
Completed SDOH surveys	29	7
White		
Total	816	65.3
Completed SDOH survey	352	85
*Ethnicity*		
Hispanic/Latino		
Total	211	16.8
Completed SDOH surveys	33	8
Non-Hispanic/Latino	999	80.0
*Current Health Insurance Coverage*	423	98.1
*Employment Status*		
Employed	174	40.4
Unemployed	261	60.6
*Own or Rent Place of Residence*		
Own	323	74.9
Rent or Other Arrangement	101	23.5
*Annual Income*		
<25,000	56	13
25,000 to 35,000	23	5.3
35,000 to 50,000	28	6.5
50,000 to 75,000	64	14.9
75,000 to 100,000	47	10.9
100,000 to 150,000	77	17.9
150,000 to 200,000	24	5.6
>200,000	47	10.9

Sample sizes (N) and percentages (%) vary based on *All of Us* Research Program available data.

**Table 3:** Odds ratio values and confidence intervals ([CI] 95% lower and upper limit) obtained by models of association between general health perception and social determinants of health.

Social Determinants of Health	OR	Lower Limit	Upper Limit	p-value

Neighborhood Characteristics	0.96	0.96	0.97	0.00
Day-to-day Discrimination	0.95	0.94	0.96	0.00
Food and Housing Security	0.57	0.49	0.67	<0.00
Spiritual Life	0.99	0.98	0.99	<0.00

P-value <0.05 is statistically significant. All the models were adjusted for gender and race.

**Table 4:** Odds ratio values and confidence intervals ([CI]95% lower and upper limit) obtained by models of association between general mental health perception and social determinants of health.

Social Determinants of Health	95% CI	Lower Limit	Upper Limit	p-value

Supportive Relationships	0.96	0.95	0.97	<0.00
Day-to-day Discrimination	0.94	0.93	0.95	0.00
Spiritual Life	0.97	0.97	0.98	0.00

P-value <0.05 is statistically significant. All models were adjusted for race and ethnicity.

**Table 5:** Odds ratio values and confidence intervals ([CL]95% lower and upper limit) obtained by models of association between social satisfaction perception and social determinants of health.

Social Determinants of Health	95% CI	Lower Limit	Upper Limit	p-value

Spiritual Life	0.98	0.97	0.98	0.00
Supportive Relationships	0.94	0.93	0.94	0.00
Food and Housing Security	0.53	0.45	0.62	<0.00
Day-to-day Discrimination	0.93	0.92	0.94	0.00

P-value <0.05 is statistically significant. All models are adjusted for race and ethnicity.

## HIGHLIGHTS

- Unfavorable social determinants of health (SDOH) conditions negatively influence the overall perception of health.
- Experiencing day-to-day discrimination increases the odds of having a poor perception of overall health.
- AA/Black and Hispanic ovarian cancer survivors have a poorer perception of overall health compared to Whites.
