# Supplementary material for: Elucidating the influences of social determinants of health on perceived overall health among African American/Black and Hispanic ovarian cancer survivors using the NIH All of Us Research Program
Source: Gynecol Oncol. Author manuscript; Available in PMC 2025 Jul 11. (PMC12247223; doi:10.1016/j.ygyno.2024.06.027)
Supplement: Supplementary Tables [file NIHMS2094845-supplement-Supplementary_Tables.docx]

**Supplementary Table 1.**

Odds ratio values and confidence intervals ([CL] 95% lower and upper limit) obtained by models of association for perception of general health and race when adjusted for social determinants of health scores and ethnicity.

| **Race** | **SDOH Variable Adjusted** | | **95% CI** | | **Lower**  **Limit** | **Upper**  **Limit** | | **p value** | |
| --- | --- | --- | --- | --- | --- | --- | --- | --- | --- |
| Black or African American (Ref: White) | | Neighborhood Characteristics | | 2.484906 | 2.094047 | | 2.949603 | | 0.00 |
| Black or African American (Ref: White) | | Day-to-day discrimination | | 1.762092 | 1.349909 | | 2.299985 | | <0.00 |
| Black or African American (Ref: White) | | Food and Housing Security | | 1.402444 | \| 1.070891 \| \| --- \| | | 1.833165 | | <0.00 |
| Black or African American (Ref: White) | | Spiritual Life | | 3.301745 | 2.444512 | | 4.459973 | | 0.00 |

Note. P-value <0.05 is statistically significant.

**Supplementary Table 2.**

Odds ratio values and confidence intervals ([CL] 95% lower and upper limit) obtained by models of association for perception of general mental health and race when adjusted for social determinants of health scores and ethnicity.

| **Race** | **SDOH Variable Adjusted** | | **95% CI** | **Lower**  **Limit** | **Upper**  **Limit** | **p value** |
| --- | --- | --- | --- | --- | --- | --- |
| Black or African American (Ref: White) | | Supportive  Relationships | 1.762092 | 1.349909 | 2.299985 | <0.00 |
| Black or African American (Ref: White) | | Day-to-day  discrimination | 1.402444 | 1.070891 | 1.833165 | 0.01 |
| Black or African American (Ref: White) | | Spiritual Life | 3.301745 | 2.444512 | 4.459973 | <0.00 |

Note. P-value <0.05 is statistically significant.

**Supplementary Table 3.**

Odds ratio values and confidence intervals ([CL] 95% lower and upper limit) obtained by models of association for perception of social satisfaction and race when adjusted for social determinants of health scores and ethnicity.

| **Race** | **SDOH Variable Adjusted** | **95% CI** | **Lower**  **Limit** | **Upper**  **Limit** | **p value** |
| --- | --- | --- | --- | --- | --- |
| Black or African American (Ref: White) | Supportive  Relationships | 1.683181 | 1.3077690 | 2.166287 | <0.00 |
| Black or African American (Ref: White) | Food and Housing  Security | 1.307630 | 0.8713608 | 1.9595889 | 0.19 |
| Black or African American (Ref: White) | Day-to-day  Discrimination | 1.143260 | 0.8893193 | 1.468138 | 0.29 |
| Black or African American (Ref: White) | Spiritual Life | 2.677184 | 2.0185924 | 3.551436 | <0.00 |

Note. P-value <0.05 is statistically significant.

**Supplementary Table 4.**

Odds ratio values and confidence intervals ([CL] 95% lower and upper limit) obtained by models of association for perception of general health and ethnicity when adjusted for social determinants of health scores and race.

| **Ethnicity** | **SDOH Variable Adjusted** | **95% CI** | **Lower**  **Limit** | **Upper**  **Limit** | **p value** |
| --- | --- | --- | --- | --- | --- |
| Hispanic or Latino (Ref: Not Hispanic) | Neighborhood  Characteristics | 2.867296 | 1.972920 | 4.184017 | <0.00 |
| Hispanic or Latino (Ref: Not Hispanic) | Day-to-day  Discrimination | 3.455379 | \| 2.028638 \| \| --- \| | 5.960456 | <0.00 |
| Hispanic or Latino (Ref: Not Hispanic) | Food and Housing  Security | 4.404646 | 1.850626 | 10.769781 | 0.00 |
| Hispanic or Latino (Ref: Not Hispanic) | Spiritual Life | 2.981430 | 1.579283 | 5.721636 | 0.00 |

Note. P-value <0.05 is statistically significant.

**Supplementary Table 5.**

Odds ratio values and confidence intervals ([CL] 95% lower and upper limit) obtained by models of association for perception of general mental health and ethnicity when adjusted for social determinants of health scores and race.

| **Ethnicity** | **SDOH Variable Adjusted** | **95% CI** | **Lower**  **Limit** | **Upper Limit** | **p value** |
| --- | --- | --- | --- | --- | --- |
| Hispanic or Latino  (Ref: Not Hispanic) | Supportive  Relationships | 8.400372 | 4.729682 | 14.832486 | <0.00 |
| Hispanic or Latino  (Ref: Not Hispanic) | Day-to-day Discrimination | 5.808410 | 3.348044 | 9.972557 | <0.00 |
| Hispanic or Latino  (Ref: Not Hispanic) | Spiritual Life | \| 5.265273 \| \| --- \| | 2.751788 | 10.001542 | <0.00 |

Note. P-value <0.05 is statistically significant.

**Supplementary Table 6.**

Odds ratio values and confidence intervals ([CL] 95% lower and upper limit) obtained by models of association for perception of social satisfaction and ethnicity when adjusted for social determinants of health scores and race.

| **Ethnicity** | **SDOH Variable Adjusted** | **95% CI** | **Lower**  **Limit** | **Upper**  **Limit** | **p value** |
| --- | --- | --- | --- | --- | --- |
| Hispanic or Latino (Ref: Not Hispanic) | Supportive  Relationships | 6.901535 | 4.421755 | 10.785150 | <0.00 |
| Hispanic or Latino (Ref: Not Hispanic | Food and Housing  Security | 5.637695 | 2.726737 | \| 11.701915 \| \| --- \| | <0.00 |
| Hispanic or Latino (Ref: Not Hispanic) | Day-to-day  Discrimination | 4.538565 | 2.944389 | 7.000352 | <0.00 |
| Hispanic or Latino (Ref: Not Hispanic) | Spiritual Life | 4.731537 | \| 2.786153 \| \| --- \| | 8.041072 | <0.00 |

Note. P-value <0.05 is statistically significant.

**Scoring for Social Determinant of Health Questionnaire Response Items**

**Neighborhood Characteristics**

These are questions for whom agreeing meant a better perception of social determinant of health:

Strongly agree = 4 , Agree =3,Neutral/ Neutral (Neither agree nor disagree) =2, Disagree =1, Strongly disagree=0

- Neighbors take good care of houses
- Neighbors share same value
- Neighbors willing to help
- Neighborhood safe
- People watch out for each other
- People can be trusted
- Neighborhood clean
- People generally get along

These are questions for whom disagreeing meant a better perception of social determinant of health:

Strongly disagree = 4 , Disagree =3,Neutral/ Neutral (Neither agree nor disagree) =2, Agree =1, Strongly agree=0

- Too many people hanging around the streets
- Too much alcohol use
- Vandalism common
- Lots of graffiti
- Lots of abandoned building
- Lots of crime
- Too much drug use
- Neighborhood noisy
- Trouble with neighbors

**Discrimination in Day to Day Life**

- Never : 5
- Less than once a year :4
- A few times a year : 3
- A few times a month : 2
- At least once a week : 1
- Almost every day : 0

**Food and Housing Security**

For questions related to amount of money to buy food:

- Never true : 2
- Sometimes true :1
- Often true : 0

For question of moving:

- 3 : 0
- <= 3 : 1

**Supportive Relationships**

- All of the time : 4
- Most of the time : 3
- Some of the time : 2
- A little of the time :1
- None of the time : 0

**Spiritual Life**

- Many times a day : 9
- Every day : 8
- Most days : 7
- More than once a week :6
- Some days : 5
- Once a week :4
- 1-3 times per month :3
- Once in a while : 2
- Less than once per month : 1
- Never/ Almost never / I do not believe in God : 0
